# Development, implementation, and evaluation of the Student Optimized Learning Environment (SOLE): a longitudinal team-based communication skills curriculum for medical students

**DOI:** 10.1186/s12909-026-08988-0

**Published:** 2026-03-14

**Authors:** Christina N. Schmidt, Nnaoma M. Oji, Karen E. Hauer, Irina V. Kryzhanovskaya

**Affiliations:** 1 School of Medicine, University of California San Francisco, San Francisco, CA USA; 2 Department of Medicine, University of California San Francisco, 1545 Divisadero St, San Francisco, CA 94115 USA

**Keywords:** Teamwork, Communication skills, Undergraduate medical education, Feedback, Curriculum development, Small-group learning

## Abstract

**Background:**

Teamwork and effective communication are essential for delivering high-quality patient care. In clinical contexts, successful teamwork depends on strong interpersonal and communication skills, making their development a critical component of undergraduate medical education.

**Methods:**

To strengthen medical students’ teamwork abilities, we developed a longitudinal, team-based communication skills curriculum. Guided by Kern’s six-step framework, we designed a small-group–based program to foster team communication, promote collaborative learning, and build feedback skills. The Student Optimized Learning Environment (SOLE) curriculum consisted of nine sessions in which students applied communication frameworks, engaged in structured reflection, and participated in case-based scenario discussions. Participants completed a curricular evaluation that included survey items and free-response questions. Quantitative data were analyzed using descriptive statistics, and qualitative comments underwent thematic analysis.

**Results:**

SOLE was implemented as a required curriculum for all medical students at a single academic medical school between 2019 and 2022. Of 174 participating students, 138 (79%) completed evaluations. Students’ responses indicated that SOLE helped them reflect on and understand team dynamics (mean = 3.74, SD = 0.9; 1 = strongly disagree, 5 = strongly agree), practice relationship-centered communication skills (3.72, 0.9), and learn (3.74, 0.9) and practice (3.69, 0.9) feedback skills. Qualitatively, students described applying skills to real-life situations, working with peers, and debriefing challenging team experiences as valued aspects of the curriculum.

**Conclusion:**

SOLE is a longitudinal, team-based communication skills curriculum aimed at fostering teamwork, feedback, and reflection within peer learning groups. Preliminary curricular evaluation indicated that SOLE offered a structured opportunity for students to engage with communication frameworks and explore strategies for effective team-based learning.

**Supplementary Information:**

The online version contains supplementary material available at 10.1186/s12909-026-08988-0.

## Background

Medicine has increasingly evolved toward more collaborative, team-based models of practice, with health care delivery now routinely involving professionals with diverse roles and responsibilities working interdependently to advance patient care [1, 2]. Within these groups of interprofessional clinicians, teamwork is vital to providing high-quality patient care [3]. High-functioning teams improve patients’ outcomes by reducing medical errors and inadvertent patient harm [2, 4–6]. Healthy team dynamics are also central to building positive working and learning environments, as team members feel greater role satisfaction and growth when working effectively with others toward a shared goal (e.g., optimal patient care) [7]. As future physicians, medical students must develop and practice the teamwork skills required to thrive in these collaborative environments.

Successful teamwork in medical contexts is rooted in strong interpersonal and communication skills. The Accreditation Council for Graduate Medical Education (ACGME) in the United States of America identifies "Interpersonal and Communication Skills” as one of the six domains of competence for physicians, specifically highlighting the ability to “work effectively with others as a member or leader of a health care team” as a key competency for physicians [8]. Deficits in these vital skills can contribute to team conflict and have been associated with adverse effects on patient care [9]. While the ability to communicate effectively and contribute to team-based care is recognized as essential for medical students and resident physicians as they progress through medical training, these skills are rarely embedded as longitudinal components of medical school curricula. Prior communication skills trainings for health professional students described in the literature range from single-episode workshops to multi-day programs [10, 11], incorporating strategies such as communication frameworks, interprofessional team-building exercises, and simulated scenarios [10, 12, 13]. Most of the published literature has described curricula with limited timeframes, decreasing the opportunities for skills practice and reinforcement. Longitudinal curricula can most readily facilitate sustained skill development through spaced practice and repetition [14, 15], and have been successful in fostering the development of medical student communication skills in patient-provider contexts [16]. Lasting improvement in team-based communication skills is similarly most likely to occur when learners engage repeatedly across diverse contexts, allowing for sustained and active practice.

Two conceptual frameworks informed the design of our team-based communication skills curriculum for medical students. The first is Social-Cognitive Learning Theory (SCLT), which posits that knowledge acquisition is shaped by observing others within social interactions and experiences, providing a foundation for understanding how students might learn from their environment and from others [17]. SCLT suggests the relevance of incorporating interactive and experiential learning into communication skills curricula, allowing medical students to observe, share observations and guidance, and to practice critical teamwork skills [18, 19]. The second is Transformative Learning Theory (TLT), which posits that deep, meaningful learning occurs when students critically examine their own experiences, challenge existing assumptions, and reframe their perspectives, leading to a transformative shift in understanding. Through structured reflection, TLT suggests that students enhance their problem-solving abilities and build confidence in new roles and relationships [20, 21]. Building on these conceptual frameworks and seeking to strengthen teamwork-centered communication skills training in our undergraduate medical education (UME) curriculum, we developed the Student Optimized Learning Environment (SOLE). Spanning the first three years of students’ medical education, SOLE helps students cultivate the skills necessary to work collaboratively in clinical and academic settings [22]. In this paper, we describe the development, implementation, and evaluation of the SOLE curriculum and suggest important considerations to guide educators interested in developing similar curricula at their institutions.

## Methods

Using Kern’s six-step framework for curriculum development, we designed a longitudinal, small-group-based course that provides students with sustained opportunities to learn, practice, and refine team-based communication skills [23]. This curriculum was designed to complement the existing UME curriculum at our medical school. The University of California, San Francisco (UCSF) School of Medicine’s (SOM) curriculum is a four-year program with three core phases. The first preclinical phase (18 months) combines science instruction with early, limited engagement in patient care experiences. During this phase, students participate in traditional lectures and are embedded in longitudinal groups of 10–12 students (called “Foundational Science (FS)” groups) in which they engage in peer-based, flipped classroom learning—a model in which core content is reviewed independently and in class time is used for collaborative application of learned concepts [24]. The second clerkship phase (12 months) focuses on core clinical rotations, and the third career-launch phase (14 months) allows students to choose electives, complete a major research or scholarly project, and prepare for residency. The SOLE curriculum spanned the first two phases of students’ medical education.

### Step 1: Problem Identification

Team-based communication skills are an important component of UME curricula. At our institution, a prior curriculum called Team Learning and Communication Skills (TLCS) introduced foundational communication concepts through seven brief sessions delivered during the first 18 months of medical school, prior to clinical clerkships. However, an internal evaluation of TLCS (2017–2018) conducted by the faculty lead of TLCS and a former TLCS student (see below) revealed opportunities for improvement: students rated the course below 3 out of 5 for both improving their understanding of team dynamics and providing opportunities to practice communication skills (1 = very dissatisfied, 5 = very satisfied; *n* = 109).

### Step 2: Targeted needs assessment

We reviewed the existing TLCS curriculum and conducted interviews with student and faculty stakeholders to guide the development of a new longitudinal team-based communication skills curriculum. One member of the study team (IK) conducted five semi-structured focus groups using role-specific interview guides. Participants included 12 medical students who had completed TLCS in the preceding two years, 12 faculty instructors for the TLCS curriculum, two course directors, and two medical school deans. Focus group questions explored perceived strengths and gaps in the existing curriculum, experiences with team-based learning and feedback, and recommendations for curricular structure and placement within the broader medical school curriculum (Appendix 1). Focus group comments were documented through detailed facilitator notes. Following data collection, two study team members (IK and a collaborator) reviewed notes across focus groups to identify recurrent ideas and shared perspectives through an inductive synthesis process. This pragmatic approach was selected to inform rapid curriculum development emerging from participant-generated perspectives rather than formal qualitative theory generation.

Students expressed a preference for small-group-based didactics and reflective exercises that would foster their growth as effective team members in complex health care systems. From focus group discussions, students highlighted the value of setting and reflecting on group norms, learning strategies for providing peer feedback, and having protected curricular time to practice communication skills. Several students requested longer sessions to dive more granularly into the topics they found most interesting and relevant (for example, sessions on providing feedback to peers). Faculty echoed the value of small-group learning based on their experiences working with students in the clinical environment and further emphasized the importance of additional sessions on (1) feedback conversations with peers and supervisors and (2) cultivating situational awareness.

Translating these needs assessment findings into actionable curricular priorities**,** we identified four guiding principles for the new curriculum: (1) promote a growth mindset for team-based learning, (2) ensure protected time to practice the process of giving and receiving feedback, (3) introduce and integrate self-assessment, reflection, and group assessment, and (4) optimize team dynamics early, revisiting these principles with spaced repetition and practice in the context of clinical experiences. These insights shaped the curriculum’s theoretical frameworks, learning objectives, and chosen instructional strategies.

### Step 3: Goals and objectives

Based on our needs assessment and prior literature emphasizing the value of longitudinal, experiential communication skills curricula [10–15], we designed SOLE as a new teamwork and communication skills curriculum. While SOLE represented a substantive redesign, select foundational elements of the former TLCS curriculum were retained, such as the introduction of the forming–storming–norming–performing framework to support group learning practices [25]. In contrast, SOLE’s primary areas of focus shifted towards cultivating a growth mindset, developing skills for giving and receiving feedback, and navigating communication in challenging team situations. The primary SOLE learning objectives were to (1) critically reflect on team performance in group-based learning environments and (2) identify and practice essential communication skills. To achieve these goals, the SOLE curriculum was implemented over the first 32 months (three years) of medical school, providing ongoing opportunities for skills development as students progressed from the preclinical to clinical learning environment.

### Step 4: Educational activities and instructional strategies

#### Peer- and case-based learning approach

Cultivating the skills needed to contribute to productive, high-functioning team-based learning environments requires students to engage in deliberate practice. Experiential learning, particularly through simulated scenarios and case-based learning, has been shown to improve communication skills in patient–provider contexts [26, 27]. Building on the strengths of case-based learning, we designed the SOLE curriculum around engaging, realistic cases—drawn from real events—that students explored through role-play to model how team interactions might unfold. Grounded in SCLT, these scenarios prompted students to both observe and participate in simulated social interactions, modeling effective and ineffective teamwork behaviors. This approach leveraged the theory’s emphasis on observational learning and reflective practice, enabling students to identify, internalize, and apply modeled skills to their own team-based interactions. All cases were inspired by the experiences of senior medical students who had completed preclinical coursework and core clinical clerkships; three senior students collaborated to review SOLE sessions and recommend potential cases for inclusion based on their past experiences working in preclinical and clinical teams. Cases were deliberately selected and refined to align with predefined SOLE learning objectives and the stage of students’ preclinical or clinical training, supporting progressive development of team-based communication skills. Senior students proposed cases mapped to session goals, which were then reviewed and refined by the faculty director of the SOLE curriculum to ensure curricular alignment.

SOLE was implemented within students’ existing peer-based learning groups (FS groups, described above). Embedding interpersonal skills training into familiar learning formats can strengthen skill acquisition by leveraging supportive group dynamics [28]. Receiving and incorporating personalized feedback is a critical component of communication skills development, and working within established small-groups of trusted near-peers may enhance students’ receptiveness to suggestions and feedback [29]. Integrating curricula into established learning groups also facilitates longitudinal skill-building, allowing for iterative growth as students encounter new environments while transitioning through different stages of their medical education [16].

#### Reflection and debrief activities

The SOLE curriculum also included activities aimed at fostering self-reflection, grounded in TLT. Students were prompted to reflect on past experiences in their preclinical and clinical learning teams at multiple points throughout the SOLE curriculum. Structured debriefing sessions provided opportunities for learners to analyze their own team interactions, consider alternative approaches, and connect these insights to their future physician practices. These reflective activities included written exercises, peer-to-peer discussions, and faculty-facilitated small-group (FS group) conversations. The goal of these debriefing activities was to encourage examination of both individual contributions and group dynamics, fostering self-awareness, adaptive problem-solving, and professional identity formation.

#### Curricular content and structure

The SOLE curriculum comprised nine sessions focused on team communication, group learning, and feedback skills. Curricular content was intentionally designed to align with pre-defined SOLE learning objectives, with sessions collectively spanning the core domains of team dynamics, feedback, growth mindset, and communication across preclinical and clinical learning environments. Session content was sequenced to increase in complexity and tailored to students’ developmental stage of training (preclinical or clinical), emphasizing the types of team-based scenarios encountered at each phase of medical school. Content was grounded in established teamwork and feedback frameworks that were revisited longitudinally across the curriculum.

Table 1 illustrates the alignment between learning objectives and curricular content, demonstrating the progression from foundational skill development in preclinical small-group settings to more complex application in clinical team contexts. Key competencies—such as giving and receiving feedback and navigating team dynamics—were reinforced through repeated sessions with increasing contextual complexity (e.g., Feedback 1.0, 2.0, and 3.0). Individual session descriptions, learning objectives, and activities are detailed in Table 1.

**Table 1:** SOLE session descriptions and learning objectives

Session Title	Description	Student Objectives	Frameworks & Resources
The First-Year Curriculum			
Session 1.1 Optimizing the Small Group Learning Environment	This session includes an introduction to a highly functioning learning environments, a review of individual learning preferences, and development of small group learning principles. Students will complete the Success Types Learning Indicator to identify their learning tendencies and perform exercises to highlight their learning preference differences. Finally, students will generate a set of explicit principles to guide their learning throughout the year	• Review the personality model of learning to identify your individual learning preferences • Describe the ways in which you can adapt your work in small group to support the learning preferences of others • Develop a set of guidelines/principles to facilitate productive small group learning	• Modified Success Types Learning Indicator [30]
Session 1.2 Master Adaptive Learner	To emphasize individual contributions to creating an optimal learning environment, students will be introduced to the Master Adaptive Learner (MAL) framework and growth mindset. After reviewing the MAL, students will apply the framework to a series of cases. Students will then review growth mindset theory and complete a written reflection and group discussion reflecting on their learning mindset in past situations	• Describe the qualities and capabilities of the master adaptive learner • Distinguish between a fixed and growth mindset in approaching feedback, successes, or setbacks	• Master Adaptive Learner Framework [31] • Growth Mindset Theory [32, 33]
Session 1.3 Feedback 1.0	During this session, students will be practicing giving and receiving peer feedback. After reviewing various frameworks for effectively giving and receiving feedback, students will reflect on past feedback experiences, identifying examples and ineffective and effective feedback interactions. Students will then craft and deliver feedback to a peer related to that student’s contributions to the small group learning environment. Students discuss these feedback experience using the growth mindset principles learned in session 1.2	• Reflect on the impact of the content, process, and mindset on reactions to feedback • Examine the components of, and various models for, effectively delivering and receiving feedback • Employ strategies for seeking, receiving, delivering, and acting upon feedback from peers	• Thanks for the Feedback. The Science and Art of Receiving Feedback Well. [34] • Ask-Tell-Ask model [35]
Session 1.4 Team Assessment 1.0	Using the Tuckman’s model from group development, students will reflect on how their learning group has evolved and consider ways to heighten team learning effectiveness. Students will consider ways to heighten their team learning effectiveness using feedback strategies and a growth mindset. They will then reflect on how their group functioning has aligned with their group learning principles; they will identify next steps to improve group function and adjust their group’s learning principles	• Describe the “Forming-Storming-Norming-Performing” (Tuckman) model of team development • Describe the applicability of the Tuckman model of group development to the progress of this year as a learning team • Assess the impact of previously established learning principles on your group’s evolution as a learning team	• Tuckman Model of Group Development [36]
Session 1.5 Team Assessment 2.0	In this session, students will return to the Tuckman stages to consider how their group has evolved final few months of first year. Using role-plays, students will consider how their group changes when a new team member enters a learning group. Students will also use role plays to explore challenging situations that may arise as a student on clinical teams that are constantly changing. Students will practice acknowledging the individual dynamics within a team and use various communication skills that help them clarify expectations or give/receive feedback	• Describe the “Forming-Storming-Norming-Performing” (Tuckman) model of team development • Distinguish your personal contribution to the group’s progression this year as a learning team • Assess the impact of curricular transitions on both team formation and your personal experience working in teams	• Tuckman Model of Group Development [36]
The Second-Year Curriculum			
Session 2.1 Feedback 2.0	A few weeks into second-year, students will revisit principles of effectively giving and receiving feedback, focusing on clinical settings. Prior to the session, students will reflect on the feedback that they received in their pre-clinical training related to group participation and team dynamics. During the session, students will review real-life cases involving feedback scenarios written by previous clinical clerkship students. Students will practice skills for building self-efficacy and situational awareness when having a feedback dialogue in a busy clinical environment	• Examine the components of, and various models for, effectively giving and receiving feedback • Employ strategies for seeking, receiving, acting upon, and delivering feedback as a team member	• Thanks for the Feedback. The Science and Art of Receiving Feedback Well [34] • Ask-Tell-Ask model [35] • Calling in, calling out (adapted from the School Reform Initiative) [37]
Session 2.2 Applying SOLE Skills to Clinical Team Learning 1.0	In this session, students will apply the communications skills that they have learned in curriculum to learning in clinical teams. Going through common challenging situations encountered in clinical practice, students will practice problem-solving by applying the communication skills learned during pre-clinical SOLE sessions. Subgroup will discuss the benefits of the various communication styles in a structured reflection	• Apply the team-learning, feedback, and communication skills developed in SOLE to the clinical environment through challenging situations commonly encountered in practice	• Thanks for the Feedback. The Science and Art of Receiving Feedback Well. [34] • Ask-Tell-Ask model [35] • Validate, Challenge and Request approach [38]
Session 3.1 Feedback 3.0	As a third-year, students will return to the SOLE curriculum to reflect on the clinical feedback they have received in clinical clerkships, identifying helpful and actionable steps for growth. Through a series of cases, students will review and practice strategies for fostering self-efficacy on the wards – identifying the right time to ask for feedback, students’ role in cultivating an optimal learning environment, and adjusting course if a clinical experience is not meeting students’ expectations	• Examine the components of, and various models for, effectively delivering and receiving feedback in time-pressured situations • Employ strategies for seeking, receiving, acting upon, and delivering feedback as a member of a clinical team	• Thanks for the Feedback. The Science and Art of Receiving Feedback Well. [34] • Ask-Tell-Ask model [35]
The Third-Year Curriculum			
Session 3.2 Applying SOLE Skills to Clinical Team Learning 2.0	In the final SOLE session, students will return to the Tuckman stages and consider the teams they have worked on during clinical clerkships. Students will bring their own experiences and highlight the stages their teams flowed through. Using cases on team dynamics, feedback scenarios, and team communication, students will practice skills that can help them productively contribute to clinical teams	• Apply team-learning, feedback, and communication skills developed in SOLE to your own or previous medical students’ challenging clinical scenarios • Recognize aspects of team dynamics in your clinical environment and commonly encountered in inter-professional practice	• Tuckman model of group development [36] • Ask-Tell-Ask model [35] • Validate, Challenge and Request approach [38] • PEARLS [39] • ISBAR [40] • Conflict Styles Assessment [41]

SOLE sessions were also designed to reinforce content from the broader UCSF SOM curriculum. For example, to align with the UCSF SOM’s anti-oppressive education objectives, all SOLE session materials underwent review by senior students, faculty members, and a curricular review committee dedicated to promoting anti-oppression in the learning environment. This collaborative process informed the development of cases and scenarios designed to challenge assumptions, promote critical thinking, and apply an equity lens. The review process was iterative, allowing for continuous refinement of the curriculum.

#### Delivery and facilitation

Each SOLE session was facilitated by a single trained faculty member who remained with the same student group across the curriculum to promote continuity and relationship-building. Prior to each session, students reviewed the learning objectives and relevant reading materials covering theoretical frameworks related to the session content. Students also completed a formal written reflection on how the topics to be explored related to their personal experiences in preclinical and clinical learning environments. During each curricular session, students first discussed the theories and frameworks relevant to the session’s content and then shared their written reflections within the small-group. Students then moved into interactive exercises centered on applying the learned content through activities including role-plays, case-based scenarios, and small-group discussions.

Curriculum facilitators participated in structured, hour-long training sessions prior to each SOLE session, led by the SOLE course director. Each preparatory hour reviewed the underlying theoretical frameworks, session-specific content, and strategies for fostering a productive group learning environment. Training not only leveraged the experience of seasoned facilitators to suggest optimal facilitation strategies but also incorporated current evidence from the science of learning and best practices for navigating sensitive discussions, modeling inclusive communication practices, and supporting equitable student participation. Consistent with the broader SOM curriculum, SOLE was graded on a pass/fail basis.

### Step 5: Implementation

**Fig. 1:**
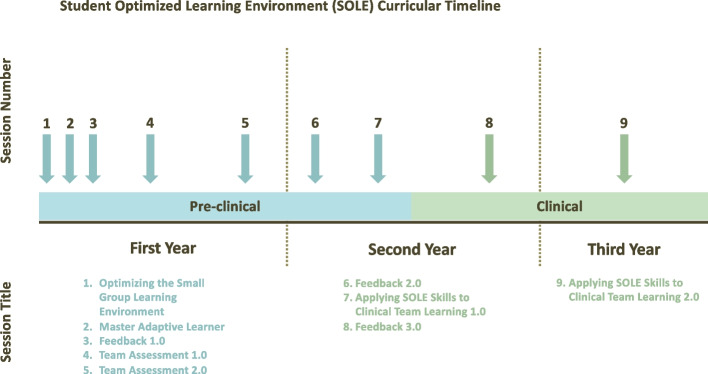
Student Optimized Learning Environment (SOLE) curricular timeline

The first iteration of the SOLE curriculum was implemented at our institution with the medical school class graduating in 2023. Students participated in nine sessions over the first three years of medical school (2019–2021) within their assigned peer groups (Fig. 1). Facilitators included SOM faculty, staff from the Office of the Ombuds, volunteer clinicians, residents, and senior students, representing the departments of surgery, medicine, neurology, psychiatry, psychology, pediatrics, pathology, radiology, biochemistry, anatomy, genetics, and behavioral sciences. Facilitators were invited to participate through the medical school’s existing instructor network and were primarily drawn from faculty and staff already engaged in other components of the undergraduate medical curriculum.

### Step 6: Evaluation

Students who participated in SOLE were invited to complete an end-of-program evaluation consisting of a seven-item survey assessing their agreement with statements about the curriculum’s effectiveness (Appendix 2). Responses were scored on a five-point Likert scale (1 = strongly disagree, 5 = strongly agree). Likert-scale responses were summarized descriptively; item-level response distributions were not available from the institutional evaluation system used for this curriculum. Participants were also invited to provide open-ended feedback through a free-response text box. Qualitative comments were analyzed using thematic analysis with an inductive approach [42, 43]. Two study team members (CS, NO) independently reviewed all comments and applied broad initial codes. The coding team (CS, EK, NO) then met to discuss and refine these codes, creating a single, consensus-based codebook. One coder (NO) applied the final codebook to all comments, and we calculated the relative frequency of comments containing each code. All qualitative and quantitative analyses were conducted in Microsoft Excel (Microsoft Corporation, Redmond, WA).

### Ethics and approvals

This study was reviewed and deemed exempt by the University of California, San Francisco Institutional Review Board (protocol #19–28,607). The need for individual participant consent was waived by the University of California, San Francisco Institutional Review Board, as the study was deemed minimal risk and part of routine educational program evaluation. All procedures involving human participants were conducted in accordance with the ethical standards of the Institutional Review Board and with the 1964 Helsinki Declaration and its later amendments.

## Results

During the first implementation of the SOLE curriculum, 174 students participated in 13 h of instruction across nine small-group sessions. Of these 174 students, 138 (79%) completed the evaluation of the SOLE curriculum. Overall, students agreed that the SOLE sessions helped them reflect on and understand team dynamics (mean = 3.74, SD = 0.9; 1 = strongly disagree, 5 = strongly agree) and gave them the opportunity to practice relationship centered communication skills in group settings (mean = 3.72, SD = 0.9). They also agreed that they learned (mean = 3.74, SD = 0.9) and consistently practiced (mean = 3.69, SD = 0.9) strategies for acting on, as well as providing and receiving, feedback (Table 2). While mean scores reflected generally positive perceptions, ratings below 4.0 indicate that students’ satisfaction, while favorable, was moderate.

**Table 2:** Student ratings of the student optimized learning environment (*n* = 138)

Survey Questions	Mean^1^
SOLE sessions helped me to reflect on and better understand team dynamics	3.74
SOLE provided opportunities to practice relationship-centered communication skills in a group setting	3.72
I learned strategies for acting on the feedback that I received during SOLE	3.70
I practiced strategies for acting on the feedback that I received during SOLE	3.61
I learned strategies for effectively providing and receiving feedback to peers and supervisors during SOLE	3.74
I practiced strategies for effectively providing and receiving feedback to peers and supervisors during SOLE	3.69
SOLE sessions helped me apply skills learned during pre-clinical training to clinical team settings	3.52

^1^ 5-point scale: 1 = Strongly disagree, 5 = Strongly Agree

Of the 138 students who evaluated the SOLE curriculum, 94 (68%) provided written feedback describing strengths of the curriculum and areas for improvement. Reported strengths included opportunities to apply practical skills to real-world situations, engage in peer-based skills practice, build relationships centered on learning, and debrief challenging team experiences. Several students also noted that SOLE enhanced their ability to navigate the clinical learning environment, with one stating, “The SOLE curriculum was helpful in allowing me to make the most of my clerkship experience,” and another commenting, “I feel the SOLE curriculum provides us with an array of tools that are incredibly helpful during clerkships.”

Common recommendations for curricular improvement focused on enhancing efficiency and timing. Students emphasized that sessions could be streamlined, with one noting, “I do feel that it could be condensed down by quite a bit and still have the same impact.” Others suggested that certain sessions occur earlier in training to better align with clinical skills and communication curricula: “Some aspects of the vignettes might have been more useful to discuss as a SOLE group prior to the beginning of clerkships, as the skills introduced in the session were already learned on the wards.” Strengths and recommendations are summarized in Table 3.

**Table 3:** Themes from qualitative analysis of the student optimized learning environment curriculum (*n* = 94)

	**Themes**	**Response number (n)**	**Representative quotes**
Strengths	Practical Skills	14	“I found myself using a lot of the communication skills that I practiced in SOLE sessions. Overall, these sessions were very useful.” “I find SOLE curriculum very helpful in terms of teaching us different techniques and strategies to communicate feedback.” “SOLE was an especially helpful aspect of [the curriculum] …that slowly introduced me to useful skills that help students thrive during clerkship learning.” “I think the most useful part of SOLE during the pre-clinical years was providing us with a framework on how to handle difficult interactions that may arise on wards”
	Peer Learning	10	“I had some good conversations with classmates about [team interactions] …sharing negative and positive experiences.” “…allowing us to break up into pairs or groups helps, as we are then able to share our experiences with each other and learn from our peers.”
	Debrief	9	“The greatest value I got from SOLE was a space to talk about conflicts we had had on the wards and how we dealt with them.” “SOLE provided a safe space to debrief on difficult experiences from clinical rotations.”
Recommendations	Streamlining content	10	“I do feel that it could be condensed down by quite a bit and still have the same impact” “I believe the information can be taught more efficiently at times, but the information is invaluable.”
	Optimizing timing	8	“There were some aspects of the vignettes posted that may have been more useful to discuss as a SOLE group prior to the beginning of clerkships as preparation, as the skills that could have been learned in the session were already learned on the wards.”

## Discussion

SOLE is a longitudinal, case-based curriculum that leverages existing, peer-based learning environments designed to engage students in teamwork-centered communication skills training. Based on early evaluation data from the first cohort to complete the curriculum, students reported engagement in content related to team dynamics, feedback conversations, and the application of strategies for ongoing learning. Qualitative data suggested that peer-based learning approaches were perceived as an effective method for learning communication skills. Opportunities to debrief challenging team experiences and reflect on personal growth within established peer learning groups were described as particularly valuable in qualitative comments.

Findings from our quantitative and qualitative analyses reflect the theoretical frameworks that guided SOLE’s curricular design. Consistent with SCLT, students described learning through observation of peers and through opportunities to practice communication skills within their established learning groups [17]. Similarly, principles of the TLT framework were reflected in students’ descriptions of reflecting on prior experiences and debriefing challenging experiences [20]. Students noted that these reflective exercises were particularly valuable components of the curriculum, especially as they transitioned into clinical learning environments.

To our knowledge, SOLE is the only longitudinal teamwork-focused communication skills curriculum for medical students described in the literature. Structuring the curriculum to span the first three years of medical school allowed for repetition and reinforcement of important concepts over time. Foundational frameworks, theories, and models were introduced early and revisited as students entered new team-based learning environments (e.g. the transition from preclinical to clinical training), providing opportunities to apply new insights and integrate prior experiences into their skills development across their medical education. This longitudinal structure was reinforced through peer learning communities and sought to create a consistent environment for reflection, feedback, and skills reinforcement. This design responds directly to evidence that longitudinal, spaced engagement supports sustained skills development for medical students, extending these principles to a unique, teamwork-focused curriculum [14, 15].

Leveraging peer relationships within existing longitudinal learning communities was a key element of SOLE’s curricular design. As previously described, qualitative analyses suggested that students valued these peer-based discussions—particularly opportunities to reflect on challenging situations and develop strategies for managing team dynamics. These findings align with the growing emphasis in UME on fostering sustained peer-learning communities [44]. As curricula increasingly incorporate these approaches, developing skills that enable students to succeed in team-based environments remains essential. These skills are also foundational during the transition from preclinical to clinical learning, as students assume new roles within clinical teams. SOLE was intentionally structured to support this transition, reinforcing communication and collaboration skills, and students described using skills gained through SOLE to navigate challenges in early clinical clerkships. Facilitating a smoother transition into the clinical environment can help students take a more active role on clinical teams, in turn supporting more effective team dynamics and contributing to better patient care [1].

Quantitative and qualitative findings provided complementary perspectives on students’ experiences with SOLE. Although students’ evaluations reflected overall satisfaction with the curriculum, mean scores below 4.0 on a five-point scale suggested that satisfaction was moderate rather than uniformly high. Qualitative feedback provided insight into possible explanations for the moderate satisfaction scores, such as perceived opportunities to improve the pacing and timing of sessions, which may have influenced overall ratings. These findings highlight specific areas for improvement of the curriculum, including optimizing session length, aligning activities more closely with concurrent coursework, and reinforcing the relevance of communication skills training throughout the curriculum. These results suggest opportunities to strengthen engagement and enhance the perceived value of SOLE within the broader medical school’s educational program.

Our findings have limitations. First, both quantitative and qualitative evaluations relied on student self-report, which may be subject to response and recall bias. Second, only aggregated summary statistics were available from the institutional evaluation system used for this curricular evaluation; item-level Likert response data were not accessible to the study team. As a result, we were unable to examine response distributions, assess potential skewness, or apply alternative approaches to quantitative reporting. The quantitative findings should therefore be interpreted as descriptive summaries and considered alongside the qualitative results. Third, the prior evaluation that informed SOLE’s redesign used a different instrument and study design, precluding direct quantitative comparison between the two curricula. In addition, although a primary goal of the SOLE curriculum was to enhance students’ success in their learning environments, we were unable to directly measure changes in academic performance, either in peer-based environments during the preclinical years or in team-based performance during the clerkship years. This curriculum was also implemented at a single institution. Dissemination to other settings, particularly those with established learning communities, will be important to assess reproducibility and generalizability. Further, data collection occurred during periods of educational disruption related to the COVID-19 pandemic, which may have influenced students’ learning experiences and perceptions of the curriculum. Finally, teamwork-focused communication skills development is multifaceted and complex, and no single curriculum can address all competencies required for robust teamwork. While SOLE addressed many important domains, some aspects of teamwork—such as context-specific team interactions and skills that develop primarily through longitudinal clinical experience—inevitably extend beyond the scope of this curriculum.

The next steps for SOLE include further student-responsive curricular adjustments. Planned changes include increasing the number of case-based discussion activities to promote more dynamic peer engagement. We will also create additional opportunities for students to reflect on prior learning experiences within their peer groups and clinical teams, and to discuss strategies for managing challenging team dynamics. This will involve encouraging students to share examples from their own experiences relevant to SOLE content, using these as a basis for discussion and debriefing. In addition, the curriculum will be streamlined and further aligned with the broader anti-oppressive curricular initiatives of the UCSF SOM to ensure consistency with existing equity and belonging values. To better understand SOLE’s impact on students’ academic and professional performance, future studies could examine the effect of SOLE’s skill-development sessions on students’ team performance, uptake of feedback, group learning skills, as well as with satisfaction, ownership, and roles within their learning environments.

## Conclusion

Building on the strengths of existing peer-based learning environments, SOLE provides structured opportunities for students to practice and reflect on communication skills across their medical education. Through its longitudinal, peer-based design, the curriculum aims to promote a growth mindset for learning, support feedback and reflection, and optimize team dynamics. While preliminary results are encouraging, additional evaluation is needed to determine the curriculum’s long-term impact on learning and team performance. As medical schools increasingly emphasize team-based models of learning, SOLE may offer a replicable framework for institutions seeking to integrate similar teamwork-focused communication skills training into their UME curricula.

## Supplementary Information

Supplementary Material 1.

## Data Availability

The datasets generated and/or analyzed during the current study are available from the corresponding author on reasonable request.

## Abbreviations

ACGME: Accreditation Council for Graduate Medical Education
FS: Foundational Science
SCLT: Social-Cognitive Learning Theory
SOLE: Student Optimized Learning Environment
SOM: School of Medicine
TLCS: Team Learning and Communication Skills
TLT: Transformative Learning Theory
UME: Undergraduate Medical Education
