# Supplementary material for: Development, implementation, and evaluation of the Student Optimized Learning Environment (SOLE): a longitudinal team-based communication skills curriculum for medical students
Source: BMC Med Educ. 2026 Mar 14;26:663. doi: 10.1186/s12909-026-08988-0 (PMC13107757; doi:10.1186/s12909-026-08988-0)
Supplement: Supplementary file 1 — Supplementary Material 1. [file 12909_2026_8988_MOESM1_ESM.docx]

**Appendix 1. Needs Assessment Focus Group Questions**

**Medical Students**

1. What do you understand to be the goal of the TLCS curriculum, and where does it fit within the broader medical school curriculum?
2. What aspects of learning within your small group have you found most challenging?
   *Prompts: communication challenges, uneven participation, managing dominant or quieter group members.*
3. What skills, if any, do you feel you developed through participation in TLCS?
4. Which aspects of TLCS did you find most useful for your future training or clinical work, and why?
5. Were there aspects of the TLCS curriculum that felt less useful? If so, which ones and why?
6. Did any components of TLCS feel redundant, either within the curriculum itself or in relation to other parts of the medical school curriculum? If so, where?
7. What communication skills do you feel you most need to improve?
   *Prompts: asking for or giving feedback, addressing supervisors, redirecting small-group discussions.*
8. How do you anticipate communication and teamwork challenges differing between the preclinical and clinical learning environments?
9. Looking back, what communication or teamwork skills do you wish you had learned prior to entering the clinical clerkships?
10. What suggestions do you have for improving the structure, timing, or content of the TLCS curriculum?

**Faculty Instructors**

1. What are strengths of the current TLCS curriculum in your opinion, and why?
2. What are areas of improvement in the curriculum?
3. Do you perceive overlap in the curriculum? If so, where, and how would you improve it?
4. What feedback have you heard from students that you would want a curriculum revamp to investigate?
5. What is one aspect of teaching optimal team or small-group learning that you wish had been included in TLCS but was limited by time or space?
6. What has worked well regarding scheduling, administrative support, and follow-up for TLCS?
7. Which TLCS session do you consider most effective, and why?
8. What would you change about TLCS?
9. Is there another place within the [School of Medicine] curriculum where TLCS might fit?

**Course Directors**

1. What are strengths of the current TLCS curriculum in your opinion, and why?
2. What are areas of improvement in the curriculum?
3. What feedback have you heard from facilitators or students that you would want a curriculum revamp to investigate?
4. What has worked well regarding scheduling, administrative support, and follow-up for TLCS?
5. Which TLCS session do you consider most effective, and why?
6. What would you change about TLCS?

**Medical School Deans**

1. As a dean, what challenges related to feedback or team learning do medical students commonly encounter during medical school, and where should these be addressed?
2. What feedback have you heard from students about TLCS that you would want a curriculum revamp to investigate?
3. What is one aspect of teaching optimal team or small-group learning that you wish had been included in the TLCS curriculum?
4. Where do you see TLCS fitting within the broader medical school curriculum?
5. Is there anything else you would want us to know about TLCS or include in future curriculum revisions?

**Appendix 2. Student Optimized Learning Environment (SOLE) Course Evaluation Survey Instrument**

**Instructions:**
Please rate the extent to which you agree with the following statements about the Student Optimized Learning Environment (SOLE) course.

**Response scale:**
1 = Strongly disagree

2 = Disagree

3 = Neutral/Undecided

4 = Agree

5 = Strongly agree

| Item | Statement |
| --- | --- |
| 1 | SOLE sessions helped me to reflect on and better understand team dynamics. |
| 2 | SOLE provided opportunities to practice relationship-centered communication skills in a group setting. |
| 3 | I learned strategies for acting on the feedback that I received during SOLE. |
| 4 | I practiced strategies for acting on the feedback that I received during SOLE. |
| 5 | I learned strategies for effectively providing and receiving feedback to peers and supervisors during SOLE. |
| 6 | I practiced strategies for effectively providing and receiving feedback to peers and supervisors during SOLE. |
| 7 | SOLE sessions helped me apply the skills that optimized our Foundational Sciences learning to the clinical teams I was a part of in Foundations 2. |
